# *C*-Terminal Clipping of Chemokine CCL1/I-309 Enhances CCR8-Mediated Intracellular Calcium Release and Anti-Apoptotic Activity

**DOI:** 10.1371/journal.pone.0034199

**Published:** 2012-03-27

**Authors:** Catherine Denis, Kathleen Deiteren, Anneleen Mortier, Amel Tounsi, Erik Fransen, Paul Proost, Jean-Christophe Renauld, Anne-Marie Lambeir

**Affiliations:** 1 Laboratory of Medical Biochemistry, Department of Pharmaceutical Sciences, University of Antwerp, Antwerp, Belgium; 2 Laboratory of Molecular Immunology, Department of Microbiology and Immunology, Rega Institute, K.U. Leuven, Leuven, Belgium; 3 Ludwig Institute for Cancer Research, Brussels Branch, Université catholique de Louvain, Brussels, Belgium; 4 Experimental Medicine Unit, de Duve Institute, Université catholique de Louvain, Brussels, Belgium; 5 StatUa Center for Statistics, University of Antwerp, Edegem, Belgium; National Jewish Health and University of Colorado School of Medicine, United States of America

## Abstract

Carboxypeptidase M (CPM) targets the basic amino acids arginine and lysine present at the *C*-terminus of peptides or proteins. CPM is thought to be involved in inflammatory processes. This is corroborated by CPM-mediated trimming and modulation of inflammatory factors, and expression of the protease in inflammatory environments. Since the function of CPM in and beyond inflammation remains mainly undefined, the identification of natural substrates can aid in discovering the (patho)physiological role of CPM. CCL1/I-309, with its three *C*-terminal basic amino acids, forms a potential natural substrate for CPM. CCL1 plays a role not only in inflammation but also in apoptosis, angiogenesis and tumor biology. Enzymatic processing differently impacts the biological activity of chemokines thereby contributing to the complex regulation of the chemokine system. The aim of the present study was to investigate whether (i) CCL1/I-309 is prone to trimming by CPM, and (ii) the biological activity of CCL1 is altered after *C*-terminal proteolytic processing. CCL1 was identified as a novel substrate for CPM *in vitro* using mass spectrometry. *C*-terminal clipping of CCL1 augmented intracellular calcium release mediated by CCR8 but reduced the binding of CCL1 to CCR8. In line with the higher intracellular calcium release, a pronounced increase of the anti-apoptotic activity of CCL1 was observed in the BW5147 cellular model. CCR8 signaling, CCR8 binding and anti-apoptotic activity were unaffected when CPM was exposed to the carboxypeptidase inhibitor DL-2-mercaptomethyl-3-guanidino-ethylthiopropanoic acid. The results of this study suggest that CPM is a likely candidate for the regulation of biological processes relying on the CCL1-CCR8 system.

## Introduction

Carboxypeptidase M (CPM, EC 3.4.17.12) is a member of the metallo-carboxypeptidases, which consist of metallo-peptidases cleaving *C*-terminal amino acids from peptides and proteins. CPM specifically removes arginines (Arg) and lysines (Lys) from the *C*-terminus of substrates. Its membrane-bound anchoring is propitious for local processing of peptide hormones at the cell surface [1]. The processing and modulation of the activity of kinins, enkephalins and anaphylatoxins quickly pointed towards an inflammatory role for CPM. This was further substantiated by the expression of CPM on differentiated immune cells such as macrophages, and up- or downregulation of CPM in inflammation, tumor and/or tumor environment (reviewed in [2]). However, the role of CPM in and beyond inflammation remains mainly undefined. Therefore, identification of proteolytic regulation of natural substrates by CPM remains crucial to unravel the function(s) of CPM. A polypeptide group to study in this context is the chemokine superfamily, which contributes to the inflammatory and immunological component of many clinically relevant pathologies [3]. Proteolytic processing affects the activity of chemokines *in vitro* and *in vivo* in different ways [4]. Besides orchestrating leukocyte chemotaxis and host inflammatory responses, chemokines can be regulators of fundamental developmental processes. Stromal cell-derived factor-1 alpha (SDF-1α/CXCL12α) is such a primordial chemokine. CXCL12α regulates hematopoiesis, lymphocyte trafficking, B-lineage cell proliferation, and angiogenesis. In serum, the rapid conversion of full length CXCL12α to CXCL12α des-Lys^68^ is mediated by carboxypeptidase N [5]. However, CPM also catalyzes the *C*-terminal truncation of CXCL12α, at least *in vitro* [6]. Loss of Lys^68^ negatively affects several functional properties of CXCL12α such as heparin and cell binding ability, cell proliferation, and chemotactic response of various cell types [5]–[7]. However, CXCR4 binding and internalization in response to CXCL12α (1–54) were unperturbed [8]. CCL1, ending *C*-terminally in –Lys^71^-Arg^72^-Lys^73^, forms another interesting chemokine target for CPM. CCL1 is secreted by monocytes, activated macrophages and T lymphocytes and attracts monocytes, (activated) Th2-differentiated T cells, and a subset of T regulatory cells *in vitro* [9]–[11]. CCL1 interacts exclusively with the CCR8 receptor [12]–[14]. Moreover, CCL1 exerts anti-apoptotic and proliferative activity on murine thymic lymphoma cell lines when exposed to dexamethasone (DEX) [15]. The rescue of BW5147 T lymphoma cells from corticoid-induced death by CCL1 occurs through CCR8-dependent activation of the RAS/MAPK pathway [16], [17]. Together with the specific CCR8 expression in lymphoid tissues (the thymus in particular), these observations point towards a more fundamental role for CCL1 in thymocytic migration and development *in vivo* [14]. CCL1 is implicated in inflammatory processes through leukocyte recruitment and inhibition of CCR8-mediated HIV infection [18]. Moreover, CCL1 could play an important role in angiogenesis, other viral, and tumoral processes [19]–[22]. Hence, like many chemokines, the role of CCL1 goes far beyond inflammation. The goal of this work was to investigate whether CPM-catalyzed *C*-terminal trimming of CCL1 affects its biological activity. The results of this study show that the removal of three amino acids from the *C*-terminus of CCL1 reduces CCR8 binding, but turns CCL1 into a more potent CCR8 agonist, resulting in, for example, increased intracellular calcium (Ca^2+^ *_i_*) release and enhanced anti-apoptotic activity.

## Results

### 1. CCL1 is an *in vitro* substrate for CPM

**Figure 1:**
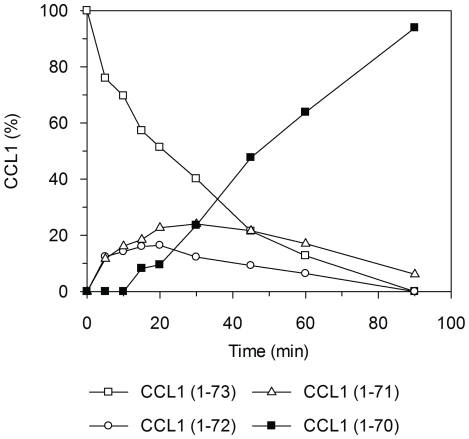
Time course of cleavage of the chemokine CCL1 by CPM. 5 µM CCL1 (R & D systems) was incubated with 26 nM CPM for various time intervals. The release of the three *C-*terminal basic amino acids –Lys^71^-Arg^72^-Lys^73^ was monitored by mass spectrometry. The percentage of CCL1 variant [CCL1 (%)] is plotted against time (min). CCL1 (1–73) (□), CCL1 (1–72) (○), CCL1 (1–71) (▵) and CCL1 (1–70) (▪).

CPM is a metallo-carboxypeptidase that cleaves off Arg and Lys from the *C*-terminus of protein and peptide substrates. We examined whether CCL1 was prone to processing by CPM. Intact CCL1 (1–73) (5 µM, produced recombinantly in *Escherichia coli*) was incubated with CPM (26 nM) at 37°C. The formation of CCL1-derived products was examined by determining the ratio of intact/cleaved polypeptide at indicated time points. Removal of Lys^73^ of CCL1 was initiated within 5 min as shown by mass spectrometry analysis (Figure 1). This step was rate-limiting and rapidly followed by the cleavage of Arg^72^ and Lys^71^. CCL1 (1–70) already appeared after 15 min. Under the conditions used, full truncation of CCL1 by CPM was achieved after 90 min at 37°C. The intermediate CCL1 products (CCL1–Lys^71^-Arg^72^ and CCL1–Lys^71^) did not accumulate. No degradation products were observed in control samples (CCL1 without CPM, 15 and 60 min of incubation at 37°C). A shorter form of the CCL1 polypeptide as well as an oxidation product were detected as minor contaminants in the original recombinant CCL1 preparation. Both products were processed identically by CPM at the *C*-terminus. The mass observed for the shorter polypeptide corresponded to CCL1 minus the five *N*-terminal amino acids Lys^1^-Ser^2^-Met^3^-Gln^4^-Val^5^ [CCL1 (6–73)]. The *k*_cat_/*K*_M_ was 2.1×10^4^ M^−1^ s^−1^ as calculated from the decay curve of intact CCL1. This value was comparable to the substrate specificity of CPM for CXCL12α [6]. Thus, CCL1 is efficiently processed at the *C*-terminus by CPM *in vitro* to generate CCL1 (1–70).

### 2. *N*-glycosylation of CCL1 slows down enzymatic cleavage by CPM

In order to investigate a potential effect of *N*-glycosylation, similar incubations (90 min at 37°C) were performed with CPM on CCL1 produced in insect cells (5 µM). The CCL1 of this preparation was susceptible to cleavage by CPM. A difference in mass of 413 Da between intact CCL1 and presumed CCL1 (1–70) was found, which corresponded to the removal of –Lys^71^-Arg^72^-Lys^73^. However, the molecular masses of CCL1 (1–73) and CCL1 (1–70) (M*_r_* of 9521 and 9108, respectively) determined by mass spectrometry were consistently higher than those predicted from the protein sequence or observed after mass analysis of CCL1 produced in *E. coli* [M*_r_* of 8484 and 8071 for CCL1 (1–73) and CCL1 (1–70), respectively]. These higher masses are presumably due to typical glycosylation that occurs during recombinant production by insect cells. Additionally, when incubated with 50 nM of CPM, only low levels of CCL1 (1–70) were detected. Yet, *N*-glycosylated CCL1 was fully converted to CCL1 (1–70) after the addition of 500 nM of CPM. The conversion was also verified by Tris-Tricine gel electrophoresis (data not shown). The relative rate of the conversion was approximatively ten times lower for glycosylated CCL1 *vs.* unglycosylated CCL1 (assuming that the initial rate is directly proportional to the concentration of CPM, with a known concentration of CCL1).

### 3. C-terminal processing of CCL1 by CPM enhances CCR8 signaling despite diminished CCR8 binding

**Figure 2:**
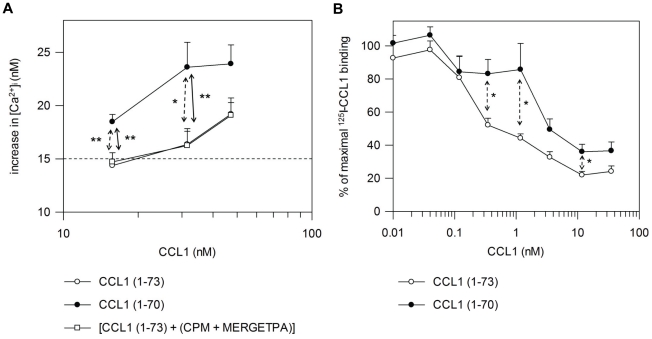
Signaling capacity through and binding properties to CCR8 of CCL1 variants. A, CHO-CCR8 cells were loaded with the ratiometric Ca^2+^-binding molecule Fura-2/AM. [Ca^2+^]*_i_* was monitored upon stimulation of the cells with the indicated concentrations of CCL1 (1–73) and CCL1 (1–70) (nM, logarithmic scale). Values represent the mean (± SEM) (nM) increase in [Ca^2+^]*_i_* (n≥6). The *dashed line* indicates the detection limit (15 nM). B, CHO-CCR8 cells were incubated with increasing concentrations of unlabeled CCL1 (1–73) or CCL1 (1–70), together with ^125^I-labeled CCL1 (1–73). The mean remaining % of ^125^I-labeled CCL1 binding (± SEM) is plotted against the concentration of unlabeled CCL1 (nM) (*n*≥5). Statistically significant differences were detected using the Mann-Whitney *U* test (*, *p*<0.05; **, *p*<0.01). Comparison of CCL1 (1–70) (•) with CCL1 (1–73) (○) is indicated with *dashed* arrows, and with [CCL1 (1–73)+(CPM+MERGETPA)] [control for CCL1 (1–73)] (□) is shown with *full* arrows.

We compared the signaling properties of CCL1 (1–73) and CCL1 (1–70) through CCR8 by measuring the Ca^2+^ *_i_* release. In CHO cells transfected with CCR8 (CHO-CCR8 cells), CCL1 (1–73) induced an increase in the intracellular calcium concentration ([Ca^2+^]*_i_*) that was dose-dependent as expected [16] (Figure 2, panel A). The Ca^2+^ *_i_* release was even more pronounced after stimulation with truncated CCL1, indicating activation of CCL1 by CPM. This increase in the release of Ca^2+^ *_i_* was statistically significant for two out of three CCL1 concentrations tested. When CPM activity was inhibited with the carboxypeptidase inhibitor DL-2-mercaptomethyl-3-guanidino-ethylthiopropanoic acid (MERGETPA) prior to the assay [control for CCL1 (1–73)], the original cellular responses were restored. Based on the dose-response curves we concluded that cleavage of CCL1 by CPM led to a significant three-fold increase of the Ca^2+^ *_i_* signaling potency. The binding efficiency of CCL1 (1–73) and CCL1 (1–70) to CCR8 was evaluated by comparing their ability to compete for ^125^I-labeled CCL1 (1–73) (Figure 2, panel B). All binding experiments were conducted in the presence of 10 µM of MERGETPA to guarantuee the inhibition of any endogenous basic carboxypeptidase activity. Both CCL1 variants competed in a dose-dependent manner for the binding to CCR8. However, truncation of CCL1 by CPM reduced the binding affinity of CCL1 (1–70) towards CCR8. The reduced CCR8 binding ability of CCL1 (1–70) was statistically significant for three CCL1 concentrations (*e.i.* 12 nM, 1.2 nM and 0.35 nM). In order to achieve a displacement of ^125^I-labeled CCL1 (1–73) comparable to that caused by CCL1 (1–73), about ten-fold more CCL1 (1–70) appeared to be necessary. ^125^I-labeled CCL1 (1–73) displacement results were comparable for CCL1 (1–73) and for the control sample for CCL1 (1–73) [CCL1 (1–73)+(CPM+MERGETPA)] (data not shown). CPM, and CPM and MERGETPA were not able to displace ^125^I-labeled CCL1 (1–73) from CCR8. Some cell samples were incubated with ^125^I-labeled CCL1 (1–73) and one of the CCL1 variants without the addition of 10 µM of MERGETPA. There was no difference in ^125^I-labeled CCL1 (1–73) displacement from CCR8 in the presence or absence of 10 µM of MERGETPA. Despite a diminished CCR8 binding efficiency, CCL1 (1–70) thus appeared to a better inducer of CCR8 signaling than CCL1 (1–73).

### 4. Significant increase in anti-apoptotic activity of CCL1 after C-terminal truncation

**Figure 3:**
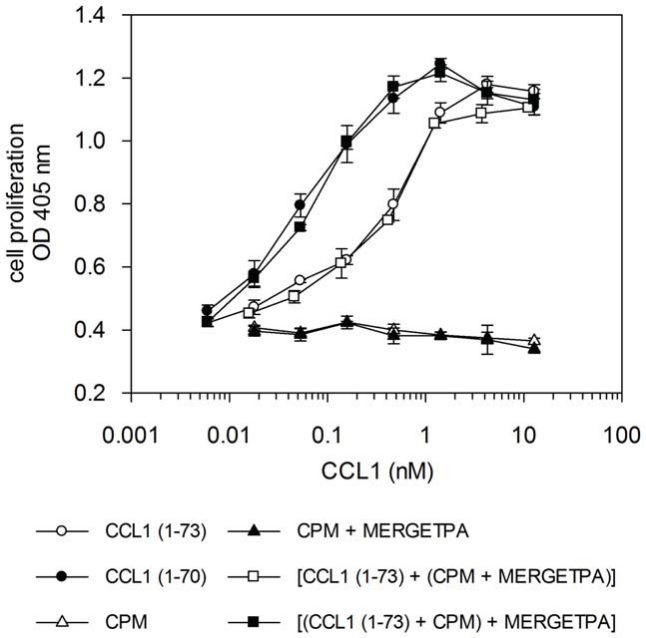
Anti-apoptotic activity of CCL1 variants on BW5147 cells. The anti-apoptotic activity of CCL1 (1–73) and CCL1 (1–70) was compared using the BW5147 cellular model. BW5147 cells were incubated with different concentrations of CCL1 (1–73) or CCL1 (1–70). Apoptosis was triggered by adding 0.25 µM DEX. After a 3-day incubation cell proliferation was determined by the colorimetric hexosaminidase assay. The OD measured at 405 nm is plotted against the concentration of CCL1 variant (nM, logarithmic scale). The graph is representative for two to three separate experiments, each performed in triplicate. CCL1 (1–73) (○), CCL1 (1–70) (•), [CCL1 (1–73)+(CPM+MERGETPA)] [control for CCL1 (1–73)] (□), [(CCL1 (1–73)+CPM)+MERGETPA] [control for CCL1 (1–70)] (▪), CPM (▵), CPM+MERGETPA (▴).

We wondered whether the increase in CCR8-mediated Ca^2+^ *_i_* release would result in an enhanced biological activity of CCL1 (1–70). The well-known BW5147 cellular model was used to compare the anti-apoptotic activities of CCL1 (1–73) and CCL1 (1–70), a process that is mediated by CCR8 [16], [17]. All the apoptosis studies all were performed in the presence of 10 µM of MERGETPA in order to inhibit any endogenous basic carboxypeptidase activity. Results are shown in Figure 3. Strikingly, an eight-fold increase in protective activity against DEX-induced death of BW5147 cells was observed for CCL1 (1–70) compared to CCL1 (1–73). Half-maximal protection was obtained at 0.47±0.07 nM and 0.06±0.01 nM for CCL1 (1–73) and CCL1 (1–70), respectively. Preincubation of CPM with MERGETPA prior to addition of CCL1 [control for CCL1 (1–73)] yielded a half-maximal protection of 0.43±0.06 nM, meaning that the original response to intact CCL1 was restored. BW5147 cells did not survive when incubated with DEX alone, DEX and CPM, or DEX, CPM and MERGETPA. Overall, these observations indicate that CPM potentiates the anti-apoptotic activity of CCL1, concomitant with the enhanced activation of CCR8 signaling by CCL1 (1–70).

### 5. BW5147 cells express the CPM transcript and show membrane-associated basic carboxypeptidase activity

CCL1 is a potent protector of T lymphoma cells against DEX-induced apoptosis. The existence of an autocrine anti-apoptotic loop in adult T cell leukemia cells (ATLs) mediated by the overexpression of CCL1 was suggested. Overexpression of CCL1 by ATLs would inhibit apoptosis in ATLs and contribute to their growth [21]. The anti-apoptotic effect of CCL1 *in vitro* was enhanced after CPM-mediated cleavage. Therefore, we wondered if the T lymphoma cells were capable of influencing the anti-apoptotic trigger of CCL1 by expressing CPM at the cell surface as a response to the presence of DEX and/or CCL1. We hypothesized that the T lymphoma cells could augment their CPM expression when in contact with DEX, thereby enhancing the possibility of the formation of truncated CCL1 if contact with CCL1 would occur. This would offer an additional mechanism for escaping DEX-induced apoptosis and would also mean that CPM could be one of the players in the process of DEX therapy resistance observed in hematological malignancies such as acute lymphoblastic leukemia. From the results described below, it appeared that the BW5147 T lymphoma cells did not respond to DEX and/or CCL1 by enhancing their CPM expression at the cell surface.

**Figure 4:**
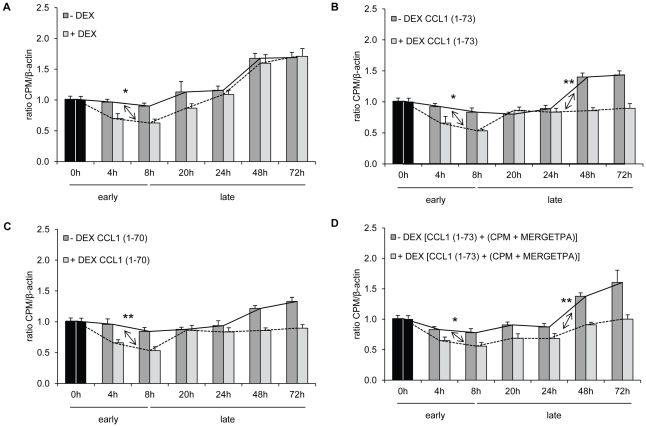
CPM transcript expression in BW5147 cells during dexamethasone-induced apoptosis. CPM mRNA expression in BW5147 cells was investigated by means of real-time PCR. BW5147 cells were incubated or not with 0.25 µM DEX and stimulated or not with 10 nM of a CCL1 variant for 72 h. CPM transcript expression was determined at the indicated time points and normalized against β-actin. The data represent the mean ratio CPM/β-actin (± SEM) plotted against time (h) obtained from three independent experiments, each performed in triplicate. BW5147 cells − DEX and BW5147 cells + DEX at time point 0 h were used as calibrators for calculations of the ratio of samples − DEX and samples + DEX respectively. Statistical differences were calculated using multiple linear regression (*, *p*<0.05; **, *p*<0.01). Arrows visualize significant differences between − DEX and + DEX on CPM transcript expression. Calibrators (black bars), samples without DEX (dark grey bars), samples with DEX (light grey bars). The stimulation condition is indicated in the upper left corner of each graph (A, no CCL1 stimulus; B, CCL1 (1–73); C, CCL1 (1–70); D, [CCL1 (1–73)+(CPM+MERGETPA)] [control for CCL1 (1–73)], respectively). To ease visual interpretation of the results, we connected the means with a line per stimulation condition (− DEX, *full* line; + DEX, *dashed* line). “Early” and “late” phases of CPM transcript expression (see *Results*, *5*.) are indicated.

Detecting CPM transcript positivity in these cells we determined whether DEX and/or CCL1 variants affected CPM transcript expression in time (Figure 4). The time course of CPM regulation occurred in two phases: (i) a decrease in CPM transcript between baseline and 8 h (early phase), and (ii) a linear increase until 72 h (late phase). Two separate statistical analyses of the data for the early and the late phase were performed, with the measurements at time point 8 h used twice. The early phase showed a downregulation of CPM, which was significantly enhanced by DEX across all CCL1 variants. This effect was independent from the CCL1 variant added. The late phase was characterized by a recovery of CPM expression across all CCL1 forms, but the recovery rate, and the influence of DEX on this recovery rate differed according to the CCL1 form. For CCL1 (1–73) and [CCL1 (1–73)+(CPM+MERGETPA)] [control for CCL1 (1–73)], DEX significantly slowed down CPM recovery. There was no significant difference in recovery rate between + DEX and − DEX for CCL1 (1–70). Finally, comparison of the rate of increase in CPM between CCL1 (1–73) with CCL1 (1–70) in the late phase showed a trend towards significance, both in the presence (*p* = 0.07) and absence (*p* = 0.05) of DEX.

**Figure 5:**
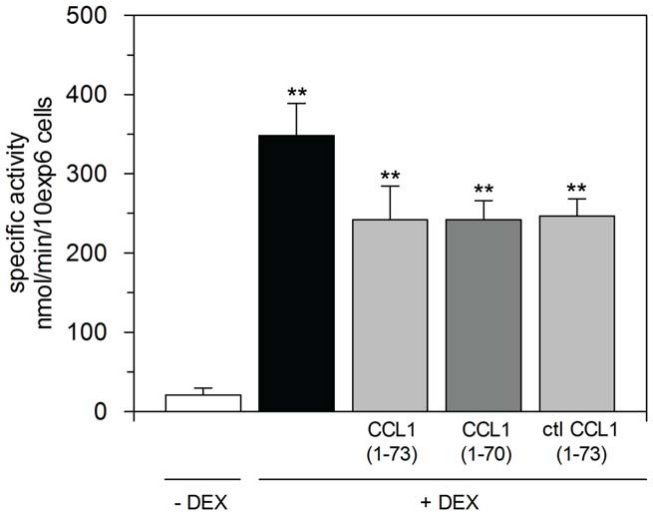
Basic carboxypeptidase activity on BW5147 cells. Basic carboxypeptidase activity was measured on intact BW5147 cells after stimulation with 10 nM CCL1 (1–73) or CCL1 (1–70) for 72 h in the presence of DEX using DAR as described in the Materials and methods section. Results represent specific activity in nmol/min/10exp6 cells (± SEM) of three independent experiments, each performed in duplicate. Statistically significant differences were detected using 2-way ANOVA analysis followed by a Dunnett test (BW5147 cells − DEX compared with conditions of BW5147 cells + DEX, **, *p*<0.01). BW5147 cells − DEX (white bar), BW5147 cells + DEX (black bar), BW5147 cells + DEX+CCL1 (1–73) or BW5147 cells + DEX+[CCL1 (1–73)+(CPM+MERGETPA)] [control (ctl) for CCL1 (1–73)] (light grey bar), BW5147 cells + DEX+CCL1 (1–70) (dark grey bar).

Membrane-associated basic carboxypeptidase activity was measured on intact BW5147 cells. Overall, the basic carboxypeptidase activity measured on BW5147 cells after 72 h of incubation with(out) DEX and/or CCL1 variants was low but still detectable (Figure 5). No difference in specific activity was seen after stimulation with CCL1 (1–73) or CCL1 (1–70) in the presence of DEX. This activity tended to be decreased compared to BW5147 cells with DEX (although not statistically proven) and correlated with the results of the CPM transcript analysis. Stimulation with CPM or CPM and MERGETPA did not influence the activity measured on BW5147 cells with DEX (data not shown). Activity on BW5147 cells without DEX was significantly lower than with DEX, or BW5147 cells with DEX and a CCL1 variant. The low activity level of BW5147 cells without DEX was not consistent with the CPM transcript level. BW5147 cells with(out) DEX showed a similar and the highest level of CPM transcript at 72 h of all conditions tested.

## Discussion

Literature data concerning natural substrates of CPM are rather scarce. Reported biologically active substrates include bradykinin [1], [23], [24], Arg^6^/Lys^6^-enkephalins [1], [23], dynorphin A (1–13) [1], epidermal growth factor [25], hemoglobin (α chain) [26], and CXCL12α [6]. For these substrates, removal of the *C*-terminal amino acid specifically modifies some of the peptides' activities (reviewed in [2]). On the other hand, post-translational modification and *N*-terminal proteolytic processing of chemokines is commonly observed and is believed to contribute to the fine-tuning of the inflammatory response [4]. In contrast to *N*-terminal proteolysis, reports describing the truncation of chemokines at the *C*-terminus are limited in number. In particular, the chemokine CXCL12α des-Lys^68^ loses heparin and cell binding ability partly, while B cell proliferation and chemotaxis are enhanced [5], [7]. In this study we demonstrate for the first time that another chemokine, CCL1, is processed at its *C*-terminus by CPM *in vitro*. CPM-mediated release of –Lys^71^-Arg^72^-Lys^73^ from CCL1 occurred very efficiently. The first step in the cleavage process [removal of Lys^73^ and (transient) formation of CCL1–Lys^71^-Arg^72^] was characterized by a *k*_cat_/*K*_M_ of 2.1×10^4^ M^−1^ s^−1^. This specificity constant is in good agreement with values reported for some of the natural substrates mentioned above. Glycosylation at Asn^29^ of CCL1 encumbered processing mediated by CPM. Since CCL1 is secreted as a glycoprotein [27] these results may be of importance for proteolytic processing *in vivo*. Obviously, it is difficult to predict whether the *N*-glycan of natural CCL1 will affect proteolytic modification since glycosylation greatly differs between insect and mammalian cells. Nevertheless, the *C*-terminus of glycosylated CCL1 still was susceptible to CPM processing.

CCL1 binds to and interacts selectively with the CCR8 receptor [12]–[14]. Following receptor activation, a rapid increase in [Ca^2+^]*_i_* is elicited that is essential for the initiation of cellular responses. CCL1-mediated Ca^2+^ *_i_* mobilization through CCR8 has been described in cells expressing the receptor endogenously, e.g. monocytes [10], HL-60 clone 15 [14], IL-2-activated natural killer cells [28], T cell lines [29], BW5147 cells [17], and U87 malignant glioma cells [30]. Release of Ca^2+^ *_i_* mediated by CCL1 has also been investigated using CCR8-transfected cells, e.g. CCR8 mouse pre-B cells 4DE4 [14], [31] and 300-19 cells [13], CCR8-transfected HEK293 cells [18], and CHO-CCR8 cells [16], [32]. In this study we compared the signaling properties of CCL1 (1–73) and CCL1 (1–70) using the CHO-CCR8 cells. As shown in Figure 2 (panel A), truncation of CCL1 by CPM potentiated the Ca^2+^ signaling capacity of CCL1. CCL1 (1–70) consistently induced higher increases of the Ca^2+^ *_i_* for the tested CCL1 concentrations. Although Louahed *et al.* reported dose-dependent Ca^2+^ *_i_* mobilization in CHO-CCR8 cells after stimulation with 0.05, 0.5 and 5 nM CCL1 [16], in other studies midpoints of dose-response curves usually lie around 1 to 5 nM CCL1, while maxima are reached at 100 nM [32]. Therefore, the concentrations we tested (range of 16 nM to 47 nM) lie in a reasonable range for Ca^2+^ *_i_* release. Remarkably, CCL1 (1–70) bound with less efficiency to CCR8 than CCL1 (1–73) (Figure 2, panel B). Chemokine receptor binding and signal transduction represent two distinct processes that usually go hand in hand. Nonetheless, weaker binding of CCL1 (1–70) to CCR8 is not exclusive for a more potent receptor activation. Concomitant with Ca^2+^ *_i_* release, activation of CCL1 was observed when comparing anti-apoptotic activity in the BW5147 model. CCL1 (1–70) protected BW5147 cells eight times better against cell death caused by DEX than CCL1 (1–73). When CCL1 truncation by CPM was blocked, a protective effect almost identical to that of intact CCL1 was seen. Together with the Ca^2+^ signaling results, this confirms that the shortened CCL1 is indeed activated by CPM. The extremely low concentration of CCL1 (1–70) needed for anti-apoptotic activity (from 0.02 nM onward) suggests that *C*-terminal proteolytic processing may be of particular significance for its biological activity.

The *N*-terminus of chemokines is regarded as extremely important for receptor interaction. *N*-terminal extension of CCL1 by one serine generates a partial CCR8 agonist [31]. It was suggested that binding to and/or activation of CCR8 could be dependent on the interaction between negatively charged residues of the receptor and positive charges of CCL1 [30], [33], [34]. However, with the elimination of the *C*-terminal tripeptide of CCL1, and therefore three positive charges, we observed a strong activation of CCR8 in spite of a reduced efficiency in CCR8 binding. One could speculate about this phenomenon. Repulsive forces between –Lys^71^-Arg^72^-Lys^73^ likely disappear after truncation. This could expose hidden residues, and perhaps facilitate their interaction with CCR8. Alternatively, a conformational change could be induced in the shortened *C*-terminal region. Another explanation for enhanced CCL1 activity could lie in the stabilization of *N*-terminal residues of CCL1 by –Lys^71^-Arg^72^-Lys^73^. Stabilization would be lost during truncation perhaps making the *N*-terminus even more flexible and accessible for receptor interaction. Of interest, it was reported that maximal binding and activity of CCL2 and CCL7 cannot be attributed solely to the *N*-terminus [35]. Alignment of the four CCR8 binding (viral) chemokines (CCL1, vMIP-I, vMIP-II and MC148) showed that the *C*-terminal –Lys^71^-Arg^72^-Lys^73^ is not conserved. However, the Lys equivalent to Lys^73^ in CCL1 is conserved in CCL2, CCL7, CCL11 and CCL15 [36]. This residue might be of importance in the interaction of *CC* chemokines with their respective receptors. Since it appeared to be the rate-limiting amino acid in the reaction with CPM, removal of Lys^73^ (or the equivalent Lys) could be the trigger for a more efficient receptor activation. To conclude, the loss of three positive charges presumably affects the binding of CCL1 to glycosaminoglycans. The presentation of CCL1 to CCR8 on the endothelial cell surface, CCL1 gradient formation, proteolytic cleavage of CCL1, and *in vivo* leukocyte migration could thus be altered [37], [38]. Two literature reports describe the interaction of CCL1 with heparin. Human CCL1 was purified on a heparin-Sepharose matrix, a standard method used for the purification of most chemokines [10]. Marro *et al.* were the first to report the dose-dependent binding of heparin to human and mouse CCL1 (dissociation constants of 150 and 100 nM, respectively) [39].

CPM transcript expression in BW5147 cells was differently regulated by DEX in function of time. Regulation of CPM transcript expression by a glucocorticoid is an interesting finding. A functional glucocorticoid responsive element was discovered in the human carboxypeptidase U (CPU) promoter. DEX increased both CPU transcript levels and promoter activity by 2-fold [40]. Interestingly, IL-6 countered the increase in CPU mRNA abundance by DEX. This effect resembles that seen in the late phase of incubation of BW5147 cells where intact CCL1 plus DEX slowed down the recovery of CPM. Modulation of CPM expression by inflammatory chemokines and anti-inflammatory glucocorticoids would fit in the inflammatory role proposed for CPM.

Basic carboxypeptidase activity was detected on BW5147 cells. Very little is known about the expression of CPM on T lymphocytes, which seems to be dependent on the cell maturation level. Only a few percentage of mature CD4+ T cells express CPM at the cell surface. Upon activation however, CPM expression is enhanced. Also, a small portion of activated CD8+ T lymphocytes gain CPM. Absence of CPM was reported for T-lineage precursor cells and most acute myeloid lymphoma samples [41]. To date, the role of CPM on T lymphocytes remains unclear. If CCL1 truncation is mediated by CPM *in vivo*, it is unlikely that T lymphocytes are the source of CPM. The T lymphoma cells that we tested did not respond to DEX and/or CCL1 by enhancing their CPM expression at the cell surface. Hence, this is likely not a mechanism by which the cells enhance their survival and proliferation capacity in the presence of apoptotic glucocorticoids such as DEX. However, this does not exclude the participation of CPM in DEX-induced resistance. CPM can be provided by numerous other cells at cancer or inflammation sites. CPM and CCR8 both have been detected on monocytes/macrophages, dendritic cells, T lymphocytes and endothelium. Alternatively, CCL1 could be proteolytically processed by CPM if present at the cell surface of CCL1-producing cells, such as bronchial epithelial cells, activated CD4+ T lymphocytes and macrophages. Importantly, CPM can be released from the cell membrane by phospholipase C action [42] and react with CCL1 in the extracellular space. Colocalization studies of CPM and the CCL1-CCR8 system would be worthwhile to investigate the possible interaction of CCL1 and CPM *in vivo*. A search for CCL1 (1–70) in tissues or biological fluids would strengthen the biological importance of the *C*-terminal truncation of CCL1.

In conclusion, removal of the *C*-terminal –Lys^71^-Arg^72^-Lys^73^ increases the anti-apoptotic activity of CCL1, which is mediated by the CCR8 receptor. Since, outside the circulation, CPM is a likely candidate for catalyzing this *C*-terminal truncation, these results warrant further investigation of the expression and activity of CPM in relation to the CCR8-CCL1 system, for instance at sites of inflammation.

## Materials and Methods

### Cell lines, chemokines and other reagents

Mouse thymic BW5147 T lymphoma cells were obtained from the American Type Culture Collection (ATCC, Rockville, Maryland, USA), and were subcloned by limiting dilution to select a clone fully susceptible to DEX-induced apoptosis, BW5147.C2 [43]. BW5147 cells were cultured in Iscove-Dulbecco's Medium supplemented with 10% fetal calf serum (FCS), 1.5 mM L-glutamine, 0.24 mM L-asparagine, 0.55 mM L-arginine and 50 µM 2-mercaptoethanol (IMDM+). CHO-CCR8 cells, kindly provided by Prof. M. Parmentier (Université libre de Bruxelles, Brussels, Belgium), were grown in F12 Nutrient Mixture (Ham), 10% FCS and 400 µg/mL G418 (complete growth medium) [16]. All culture media were from Invitrogen (Carlsbad, California, USA). Recombinant human CCL1 produced in *E. coli* was purchased from R & D Systems (Abingdon, Oxon, UK). Recombinant human CCL1 produced in insect cells with a baculovirus expression system was a gift from Prof. J. Van Snick (The Ludwig Institute for Cancer Research, Brussels, Belgium). Recombinant CPM was purified from *Pichia pastoris* supernatans as described by Deiteren *et al.* [44]. The carboxypeptidase inhibitor MERGETPA was obtained from Calbiochem (San Diego, California, USA). Murine IL-9 was produced by expression in baculovirus and purified by affinity chromatography at the Ludwig Institute for Cancer Research [43].

### Hydrolysis of CCL1 by CPM *in vitro*

Cleavage of CCL1 by CPM *in vitro* was determined by mass spectrometric analysis. 5 µM CCL1 (R & D systems) was incubated with 26 nM CPM at 37°C in 0.1 M HEPES, pH 7.4, 100 µg/mL bovine serum albumin. At several time points, aliquots were taken and quenched by addition of 1% trifluoroacetic acid solution. For CCL1 produced in insect cells, 5 µM was incubated with 400–500 nM of CPM. C18 Zip Tips (Millipore Corp., Billerica, Massachusetts, USA) were used to desalt the samples. Elution was performed progressively with 20 µL of 30% acetonitrile and 10 µL of 50% acetonitrile in 0.1% acetic acid. Analysis of the resulting mixture was performed on an Esquire ESI Ion Trap mass spectrometer (Bruker, Bremen, Germany). The instrument was used in a scan range from 450 to 1700 m/z and optimized on a m/z value near the most abundant ion of the intact polypeptide (849 or 953 m/z). After deconvolution of the spectra, concentrations of the intact and cleaved polypeptides were calculated from their relative abundance. The abundance cut-off was established at 5%. The catalytic efficiency *k*_cat_/*K*_M_ of the reaction was determined from the decay curve of intact CCL1.

To compare the activities of CCL1 (1–70) with CCL1 (1–73), CCL1 (1–70) was generated by incubating 5 µM of CCL1 (1–73) produced in insect cells with 500 nM CPM for 90 min at 37°C. The controls for (i) CCL1 (1–73) and (ii) CCL1 (1–70) were obtained by incubating CPM with 10 µM of MERGETPA for 15 min at 37°C (i) prior to the 90 min-incubation with CCL1 (1–73) [CCL1 (1–73)+(CPM+MERGETPA)], or (ii) after the 90 min-incubation with CCL1 (1–73) [(CCL1 (1–73)+CPM)+MERGETPA].

### Signaling capacity through CCR8

CCR8 signaling induced by CCL1 variants was tested by means of Ca^2+^ *_i_* release using CHO-CCR8 cells. CCR8 expression was confirmed by flow cytometry using an Allophycocyanin-labeled monoclonal anti-human CCR8 antibody (R & D systems). CHO-CCR8 cells (10^7^/mL) suspended in complete growth medium were loaded with 2.5 µM Fura-2/AM (Molecular Probes, Invitrogen, Merelbeke, Belgium) and 125 µM Probenecid (ICN Biomedicals Inc, Aurora, Ohio, USA) for 30 min at room temperature. Cells were washed and resuspended to 10^6^ cells/mL in Hanks' balanced solution with 1 mM Ca^2+^ and 0.1% (v/v) fetal bovine serum, buffered with 0.01 M HEPES/NaOH to pH 7.0. Cells were kept on ice and preincubated for 10 min at 30°C prior to the addition of the first stimulus. Fura-loaded CHO-CCR8 cells were stimulated with 16 nM, 32 nM or 47 nM of CCL1 (1–73) or CCL1 (1–70) at 30°C. In another experiment, CPM activity was abolished through pretreatment with 10 µM of MERGETPA at 37°C for 15 min [control for CCL1 (1–73)]. Excitation wavelengths were 340 and 380 nm; the fluorescence intensity ratio (R) of Fura-2 was continuously measured at 510 nm in a LS50B luminescence spectrophotometer (PerkinElmer). 50 µM digitonin and 0.01 M EGTA in 0.02 M Tris (pH 8.5) were used for the determination of *R*_max_ and *R*_min_ values. [Ca^2+^]*_i_* were calculated using the Grynkiewicz equation [45].

### Competitive CCR8 binding assay

The binding capacity of CCL1 (1–73) and CCL1 (1–70) was determined through the measurement of competition with ^125^I-labeled CCL1 (1–73) (PerkinElmer). CHO-CCR8 cells (2×10^6^), suspended in binding buffer [0.05 M HEPES, pH 7.2 containing 1 mM CaCl_2_, 5 mM MgCl_2_, 0.1% (w/v) BSA and 0.5% NaN_3_], were incubated on ice for 2 h with [^125^I]-CCL1 and varying concentrations of unlabeled CCL1 (1–73) or CCL1 (1–70). Incubations were performed in the presence of 10 µM of MERGETPA to ensure basic carboxypeptidase inhibition [present in the CCL1 (1–70) sample or endogenously on the CHO-CCR8 cells]. After incubation, the cells were washed twice with 0.9 mL of binding buffer before determination of the gamma radiation in a gamma counter. Maximal [^125^I]-CCL1 binding to CCR8 was determined in the absence of competing unlabeled chemokine and was set at 100%. Results are expressed as the percentage of remaining bound [^125^I]-CCL1.

### Dexamethasone-induced apoptosis assay

3×10^3^ BW5147 cells per well were added in a 96-well plate and stimulated with various concentrations of CCL1 variants in the presence of 0.25 µM DEX (Sigma Aldrich, Oakville, Ontario, Canada). Each concentration point was analyzed in triplicate. Following a 3-day incubation at 37°C and 5% CO_2_ cell proliferation was determined using the hexosaminidase assay [46]. Alternatively, cell proliferation was assessed using Prestoblue™ Cell Viability Reagent (Invitrogen). All experiments were conducted in IMDM+ containing 10 µM of MERGETPA to ensure the inhibition of basic carboxypeptidase activity present in medium or on the BW5147 cell surface. Murine IL-9 was used as a positive control for anti-apoptotic activity (The Ludwig Institute for Cancer Research).

### CPM expression in BW5147 T lymphoma cells

CPM expression in BW5147 cells during DEX-induced apoptosis and rescue by CCL1 was assessed with the assays described below in a) and b). BW5147 cells were stimulated with 10 nM intact or truncated CCL1 with(out) 0.25 µM DEX at indicated time points. Cell proliferation was verified systematically as mentioned in *“Dexamethasone-induced apoptosis assay”*.

#### a) CPM transcript expression in BW5147 cells

Total RNA was extracted from BW5147 cells using the InviTrap® Spin Cell RNA Mini Kit (Isogen Life Sciences, PW De Meern, The Netherlands). cDNA was generated using the High Capacity cDNA Reverse Transcription Kit following the manufacturer's protocol (Applied Biosystems, Foster city, California, USA). cDNA was stored at −20°C until analysis. Quantitative real-time PCR was performed with a ready-to-use Taqman assay targeting the mouse CPM gene (Mm01250802_m1). β-actin was used for normalization and relative mRNA quantification (Mm00607939_s1) (Applied Biosystems). Real-time PCR reaction mixtures were prepared in qPCR MasterMix (Eurogentec, Seraing, Belgium) and subjected to a standard PCR protocol (50°C for 2 min, 95°C for 10 min, and 45 cycles at 95°C for 15 s and at 60°C for 1 min) on the ABI Prism 7000 Sequence Detection System (Applied Biosystems). The relative expression ratio of transcript was computed based on its real-time PCR efficiency (E) and the cycle threshold (Ct) value of the unknown sample versus a calibrator sample ΔCt (mean_calibrator_−mean_sample_). E was calculated using the equation E = 10exp(−1/slope), applied to a dilution series of a randomly chosen sample ranging from 0 to 100 ng cDNA in triplicate. The slope of the efficiency curve was determined by plotting the logarithm of the amount of cDNA against the Ct value. Results were calculated using the equation ratio = [E_target_^ΔCt (mean^_calibrator_^−mean^_sample_^)^]/[E_reference_^ΔCt (mean^_calibrator_^−mean^_sample_^)^] according to the Pfaffl method [47]. Amplicons of 81 bp (CPM) and 115 bp (β-actin) were detected on a 2% agarose gel stained with ethidium bromide and visualized under UV-light.

#### b) Basic carboxypeptidase activity assay

Basic carboxypeptidase activity on BW5147 cells was measured using dansyl-Ala-Arg (DAR) as described [48]. Treatment of the BW5147 cells with DEX and/or CCL1 variants was performed identically as described in *“Dexamethasone-induced apoptosis assay”*. After stimulation, cells were collected by centrifugation, washed with ice-cold phosphate-buffered saline and concentrated (8X) prior to incubation with 0.2 mM DAR for 1 h at 37°C (reaction volume of 125 µL).The reaction was stopped with 1 M citrate buffer, pH 3.1. After centrifugation, supernatant was transferred in a glass tube. The product dansyl-Ala was extracted with 600 µL chloroform. Fluorescence in the chloroform layer was measured with a spectrofluorimeter (RF-5000, Shimadzu, Duisburg, Germany) (λ_ex_ 352 nm, λ_em_ 483 nm). Activity was calculated using a standard curve of dansyl-Ala and expressed in nmol/min of dansyl-Ala generated during the reaction. Results were normalized for the number of proliferating cells present (determined with Prestoblue™ Cell Viability Reagent). Inhibition tests with 10 µM MERGETPA prior to incubation with DAR were used to calculate the percentage of measured activity originating from basic carboxypeptidases.

### Statistical analysis

Statistical analyses were carried out with SPSS 16.0 software (SPSS Inc., Chicago, Illinois, USA). The results are presented as the mean ± SEM. Differences were considered significant at *p*<0.05. The applied statistical tests are specified in the figure legends.
